# Supplementary figures and images for: Roles of P-body factors in Candida albicans filamentation and stress response
Source: PLoS Genet. 2025 Mar 17;21(3):e1011632. doi: 10.1371/journal.pgen.1011632 (PMC11975087; doi:10.1371/journal.pgen.1011632)

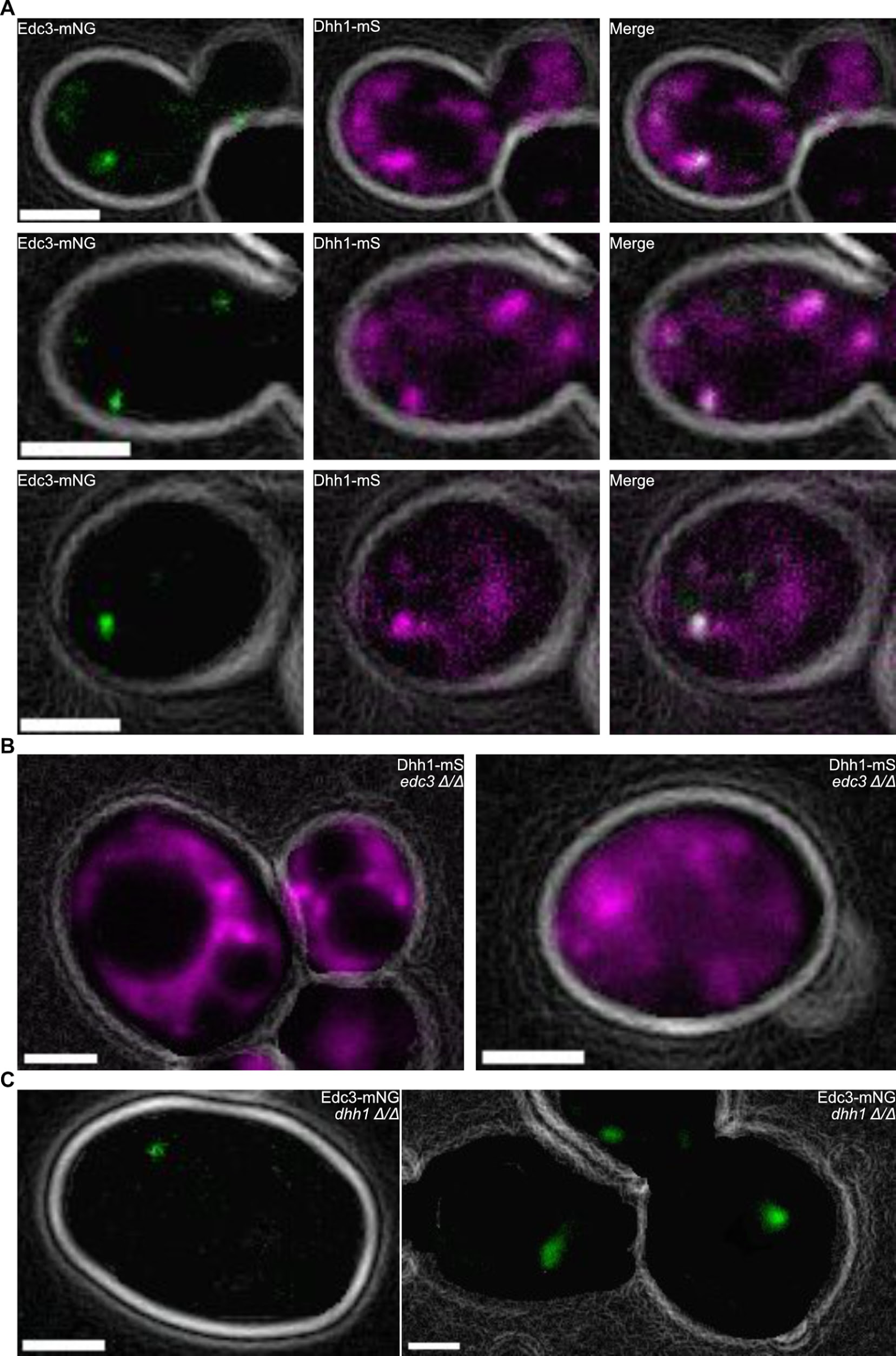

Supplement: S1 Fig — A. PB factors (Edc3-mNG and DHH1-mS) co-localized during acute heat shock (10 minutes at 46°C in CM) confirming their identity as PBs. Scale bars = 2µm. B. Dhh1-mS condensed in the absence of Edc3 in response to acute heat shock (10 minutes at 46°C in CM). Scale bars = 2µm. C. Edc3-mNG condensed in the absence of Dhh1 response to acute heat shock (10 minutes at 46°C in CM). Scale bars = 5µm. (TIFF) [file pgen.1011632.s001.tiff]

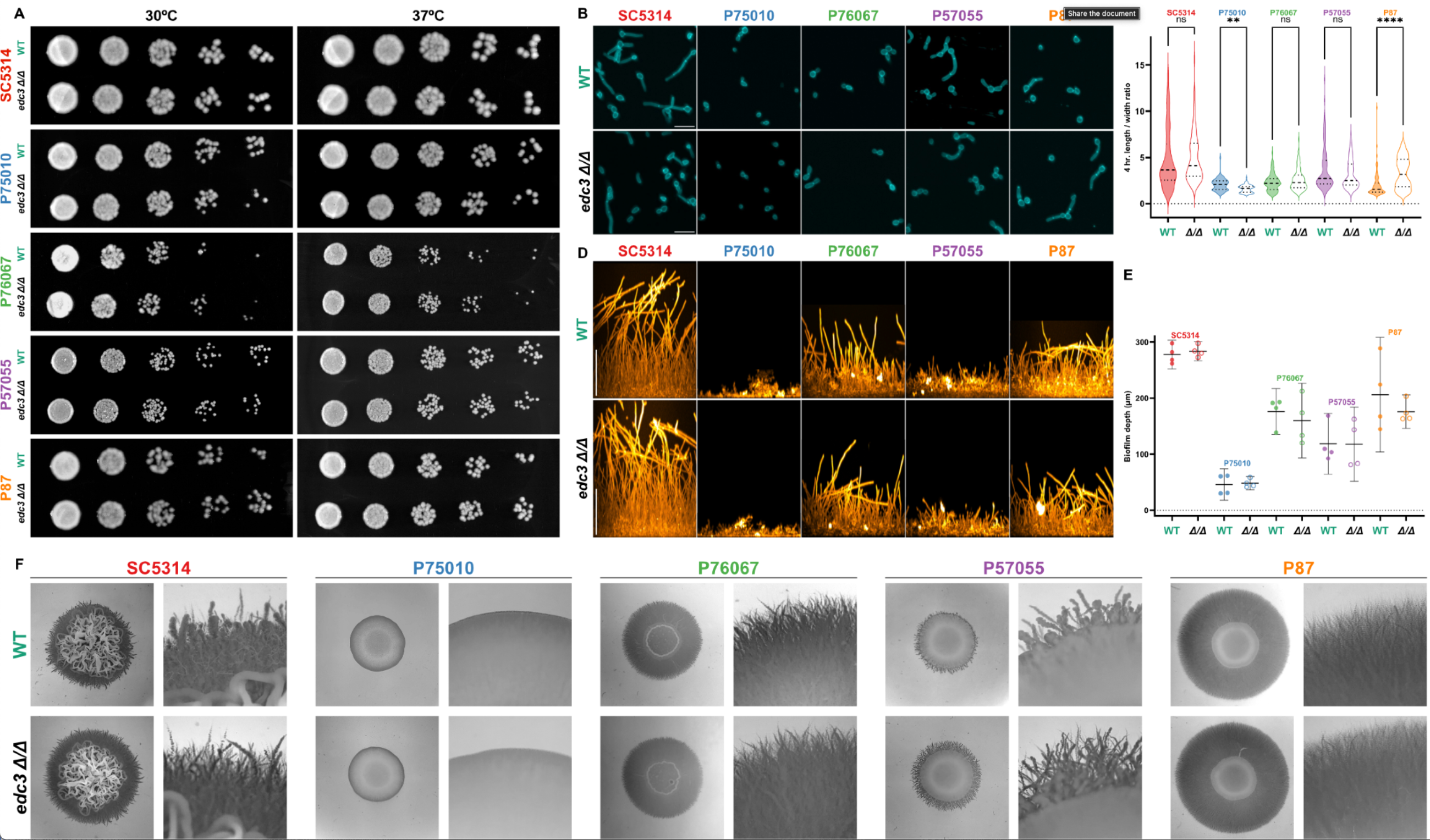

Supplement: S2 Fig — WT and edc3Δ/Δ strains were subjected to growth, filamentation, biofilm, and spider plate assays. A. 24-hour spot plate assay of C. albicans WT and edc3Δ/Δ shows EDC3 is not required for WT growth in all 5 strains. B. edc3Δ/Δ strains were subjected to a planktonic 4-hour filamentation assay (37°C, RPMI + 10% serum) and did not display gross inhibition of filamentation compared to WT. Scale bars = 20µm. C. After 4-hours of filamentation, edc3Δ/Δ did not have grossly impaired germ tube development. Only edc3Δ/Δ P75010 was significantly less hyphal than WT (N =100 cells, ****P < 0.0001, **P < 0.01, Kruskal-Wallis tests). D. Side-view of WT and edc3Δ/Δ strains after a 24-hour biofilm assay (37°C, RPMI + 10% serum). Scale bar = 100µm. E. Depth of 4 replicate biofilms. 95% CI. F. Colonies grown on spider plates for 6 days at 37°C. 6X and 25X magnification. (TIFF) [file pgen.1011632.s002.tiff]

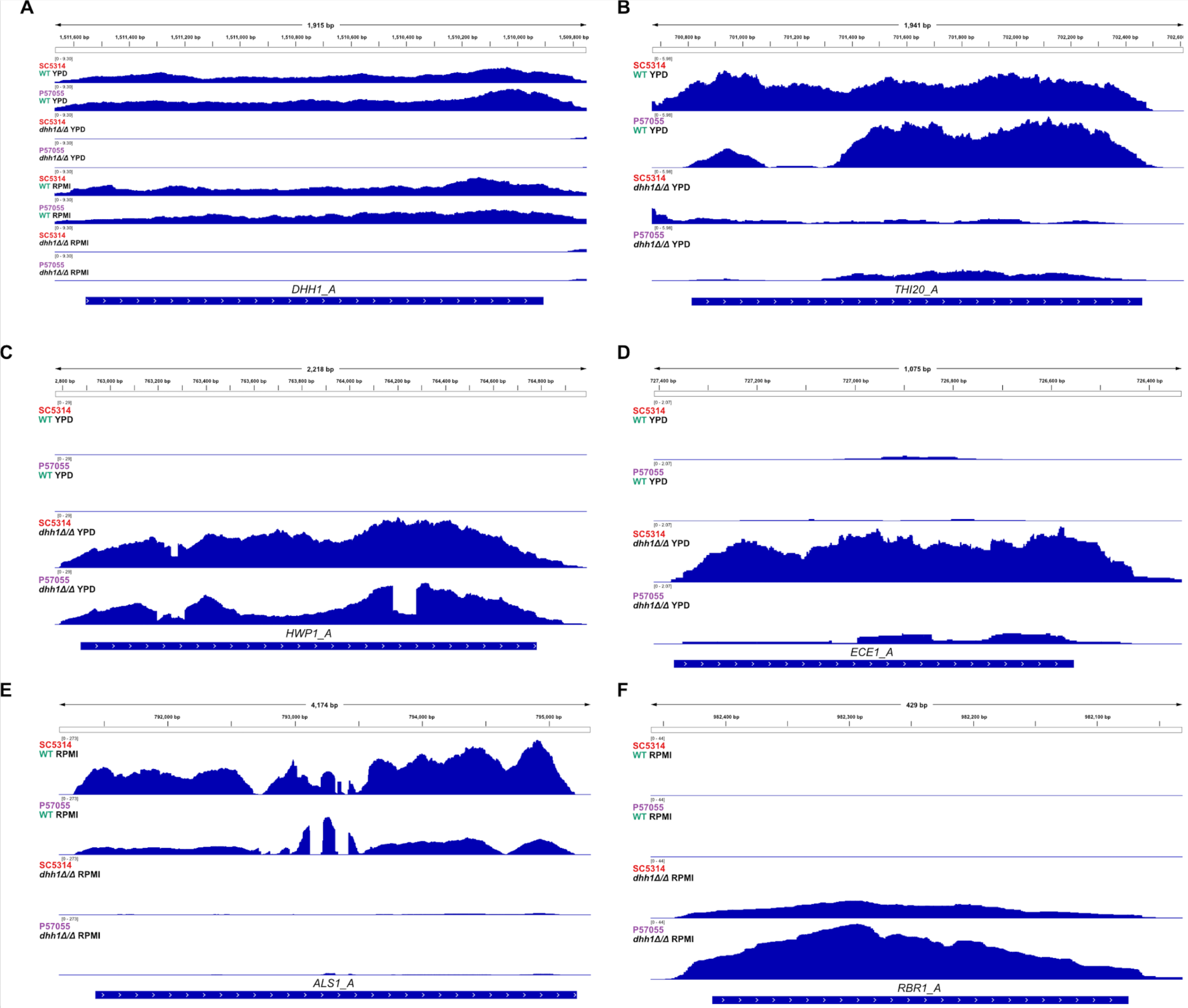

Supplement: S3 Fig — Tracks normalized by cpm were examined to verify deletions and differentially expressed genes A. DHH1 transcripts are present in WT backgrounds and absent from dhh1Δ/Δ. B. In YPD putative trifunctional thiamine biosynthesis enzyme THI20 is downregulated in both dhh1Δ/Δ strains. C. Hyphal wall protein (HWP1) transcripts are upregulated in dhh1Δ/Δ compared to WT under yeast-form growth conditions. D. Interestingly, in YPD hyphal associated candidalysin (ECE1) is upregulated in SC5341 dhh1Δ/Δ, and to a lesser extent in P57055 dhh1Δ/Δ. E. Hyphal adhesin (ALS1) is downregulated in both dhh1Δ/Δ strains in RPMI, F. while GPI-anchored cell wall protein (RBR1) is upregulated in both dhh1Δ/Δ strains under these conditions. (TIFF) [file pgen.1011632.s003.tiff]

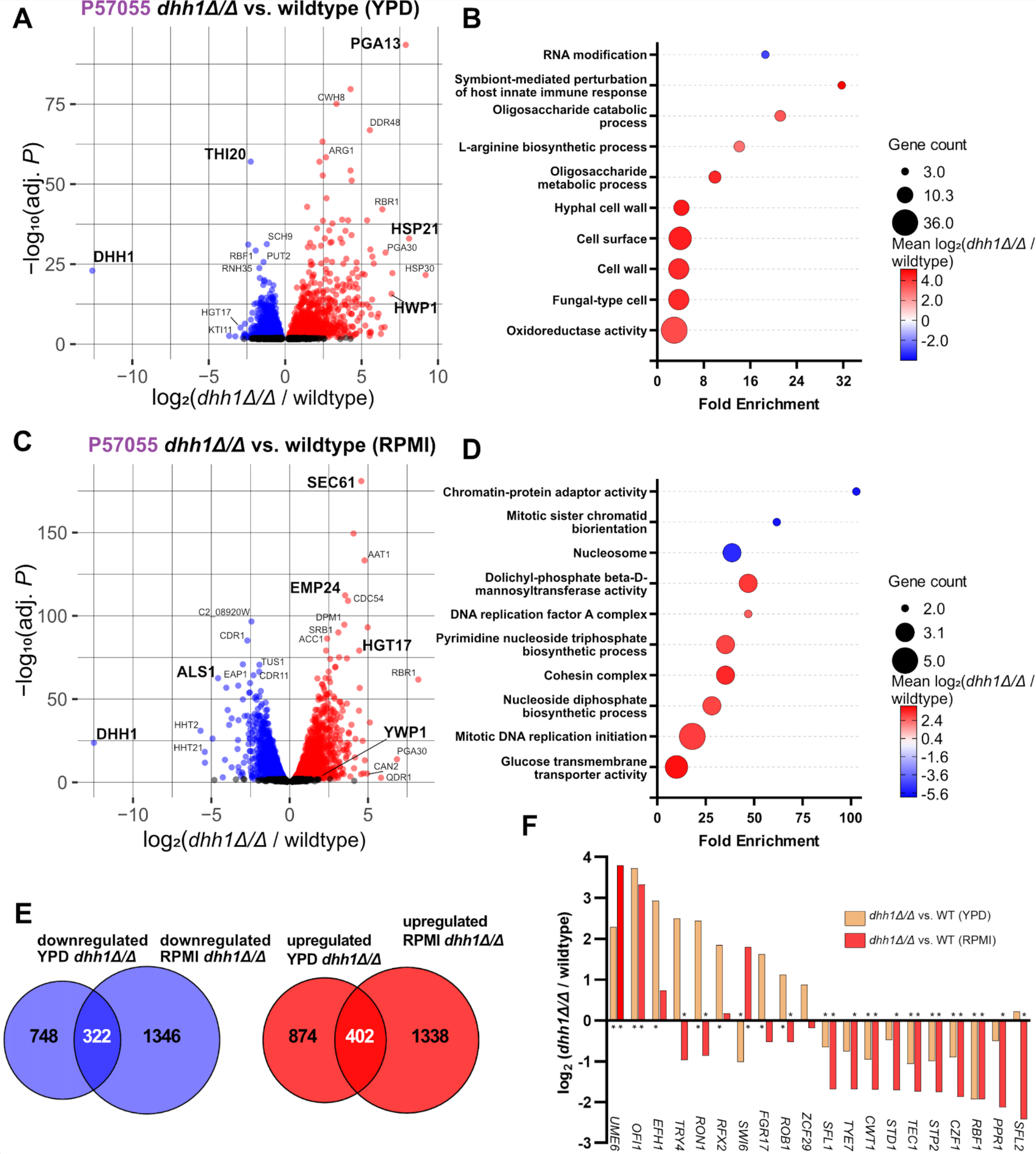

Supplement: S4 Fig — A. Volcano plot depicts genes significantly up (red) and down (blue) -regulated during yeast-form growth compared to wildtype (Padj < 0.01). Genes associated with filamentation stress were prominently upregulated (S5 Table). B. GO terms enriched in dhh1Δ/Δ YPD include transport and cell wall terms (Padj < 0.1). in. C. Volcano plot depicts gene expression differences in dhh1Δ/Δ vs wildtype during early hyphal growth (S6 Table, Padj < 0.01). D. GO terms enriched in dhh1Δ/Δ RPMI are related to chromatin, replication and biosynthesis (Padj < 0.1). E. A significant number of transcripts were downregulated (P = 0.041), and upregulated (P = 0.0178), respectively, in dhh1Δ/Δ in YPD and RPMI conditions (Hypergeometric tests). However, most differentially expressed genes were environmentally specific. F. Differential expression of transcription factors regulating morphogenesis in dhh1Δ/Δ in YPD and RPMI. (* indicates Padj < 0.01; DEseq2). (TIFF) [file pgen.1011632.s004.tiff]

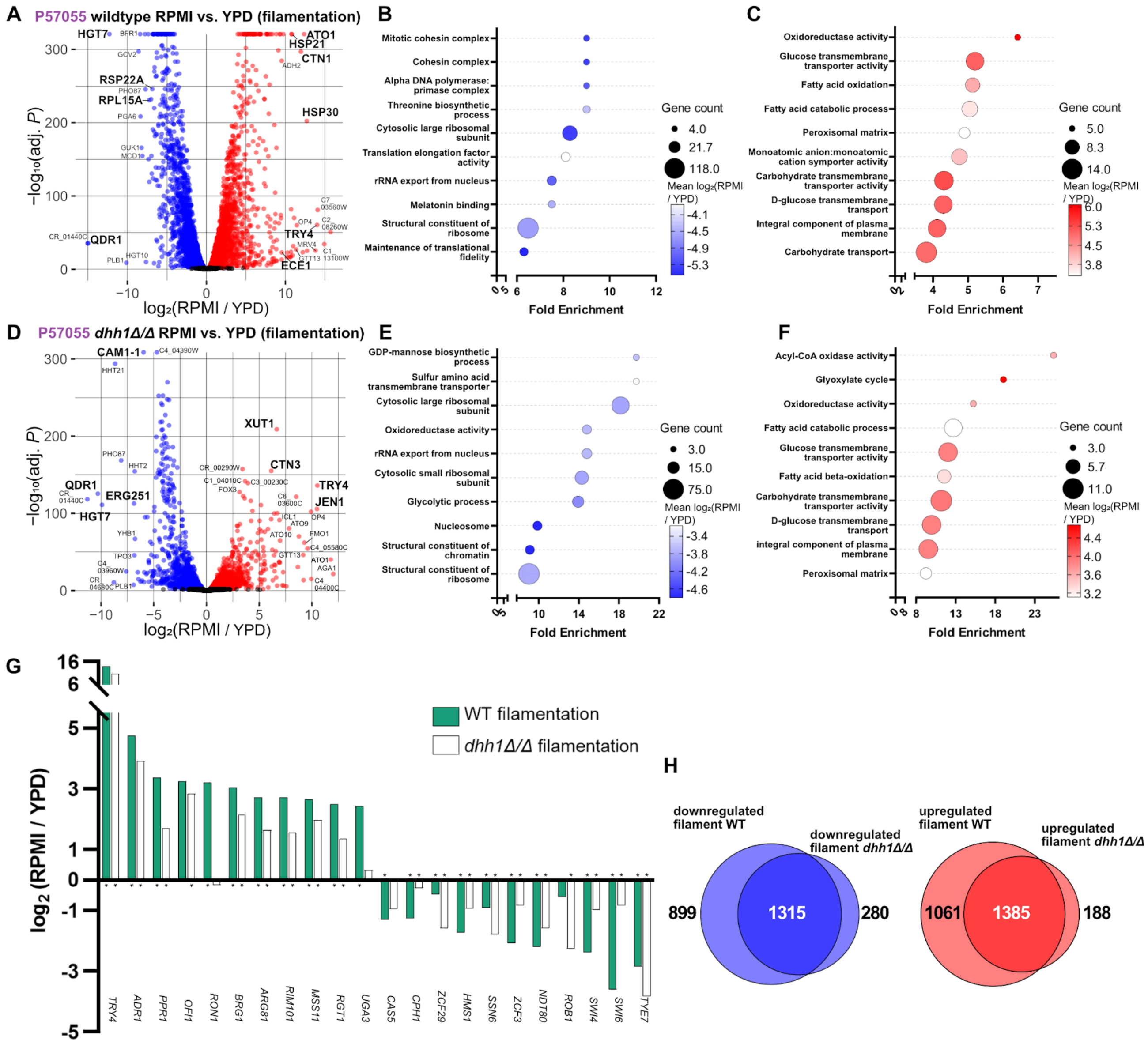

Supplement: S5 Fig — A. Volcano plots comparing the transcriptomes of WT P57055 grown in YPD and RPMI demonstrate extensive remodeling during filamentation (S7 Table, Padj < 0.01). B. Expected GO terms were downregulated, and C. upregulated during WT filamentation (Padj < 0.1). D. Comparing the transcriptome of dhh1Δ/Δ grown in YPD and RPMI showed transcriptome remodeling during filamentation (S8 Table, Padj < 0.01). E. Similar GO terms were downregulated, and F. upregulated in filamenting dhh1Δ/Δ and WT (Padj < 0.1). Unlike WT, dhh1Δ/Δ upregulated terms related to lipid catabolism during filamentation. G. Transcription factors regulating morphogenesis were differentially expressed during filamentation in WT and dhh1Δ/Δ (* indicates Padj < 0.01; DEseq2). H. Genes differentially expressed during filamentation overlapped between WT and dhh1Δ/Δ strains. Most transcripts differentially expressed during dhh1Δ/Δ filamentation are subsets of the WT remodeling response. A significant number of transcripts were down- (P = 0) or upregulated (P = 0) in both filamenting dhh1Δ/Δ and wild type (Hypergeometric tests). (TIFF) [file pgen.1011632.s005.tiff]

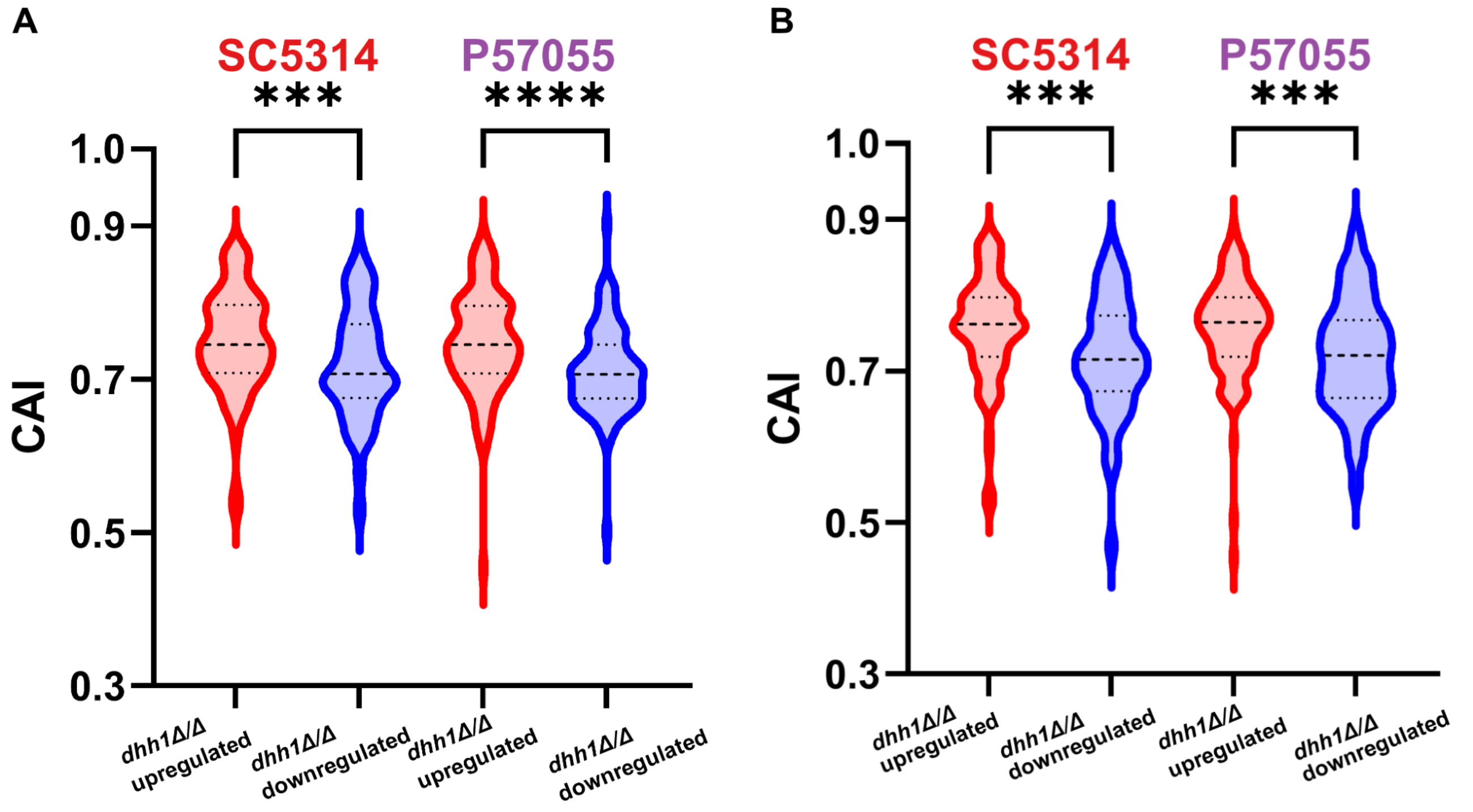

Supplement: S6 Fig — Upregulated genes in C. albicans dhh1Δ/Δ have higher CAI values than downregulated genes. CAIs of 100 most upregulated and downregulated genes in SC5314 dhh1Δ/Δ and P57055 dhh1Δ/Δ strains grown in A. YPD at 30°C and B. RPMI + 10% FBS at 37°C. (Mann-Whitney tests. ***P < 0., ****P < 0.0001). (TIFF) [file pgen.1011632.s006.tiff]

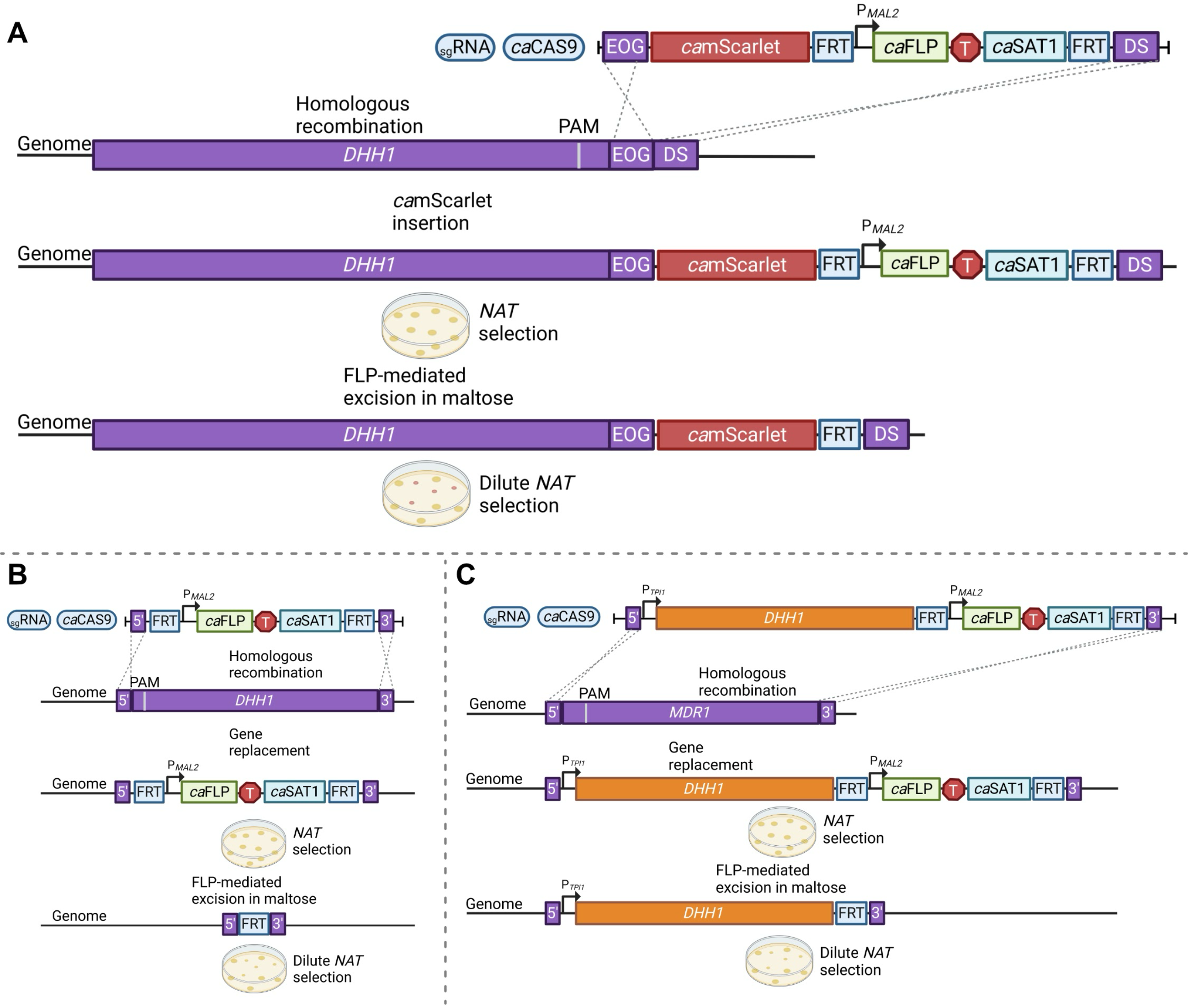

Supplement: S7 Fig — Methods for A. inserting a fluorescent tag in the genome, B. deleting the entire ORF for a gene leaving only a 34 bp FRT scar, and C. generating a DHH1 complement at the MDR1 site. Diagram produced using Biorender. (TIFF) [file pgen.1011632.s007.tiff]

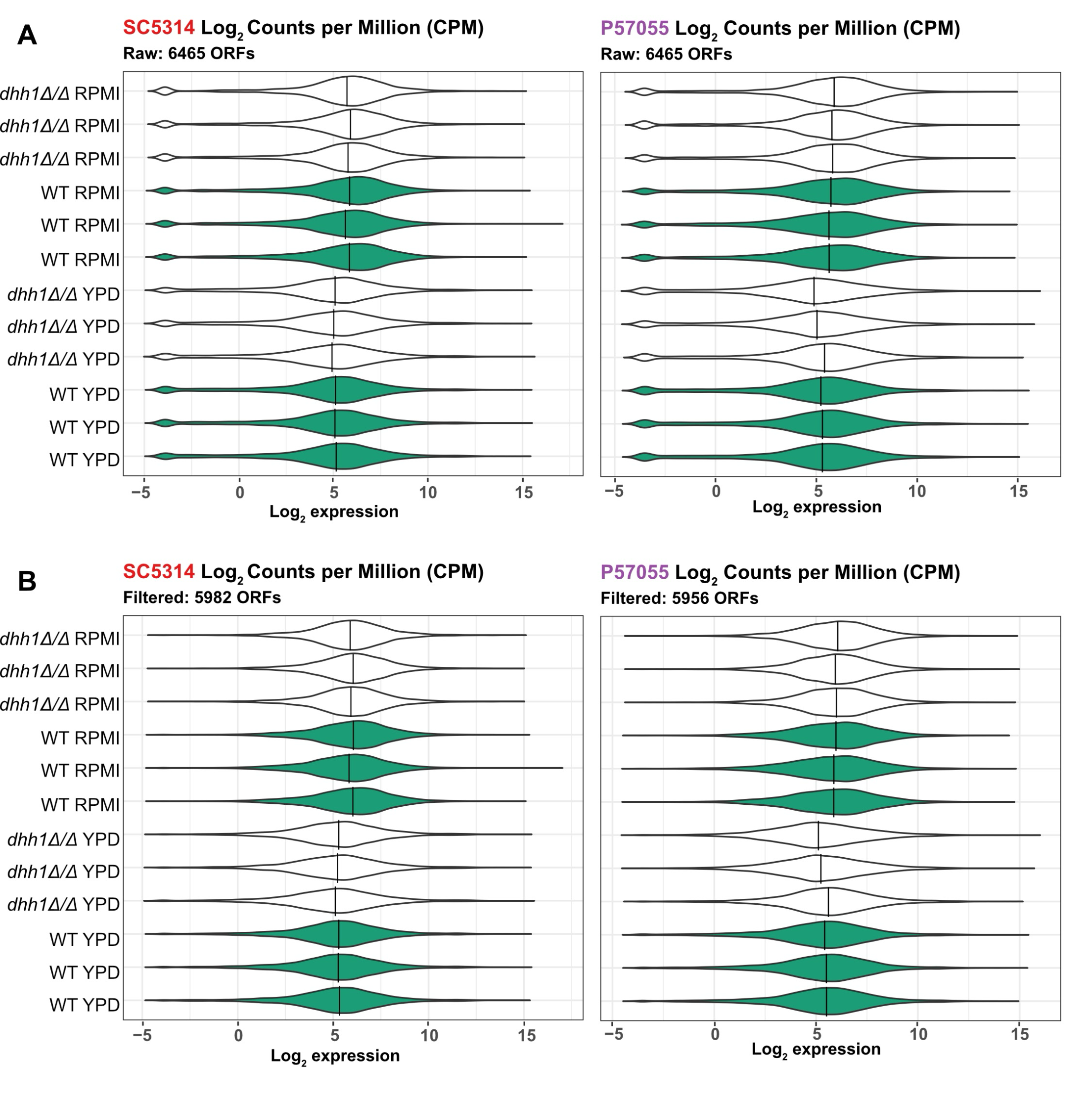

Supplement: S8 Fig — ORFs with less than one CPM hit in less than 3 experimental groups were removed prior to running DEseq2. A. Violin plots show raw and B. filtered hits for each strain and condition with ≥1 CPM in ≥ 3 experimental groups. (TIFF) [file pgen.1011632.s008.tiff]

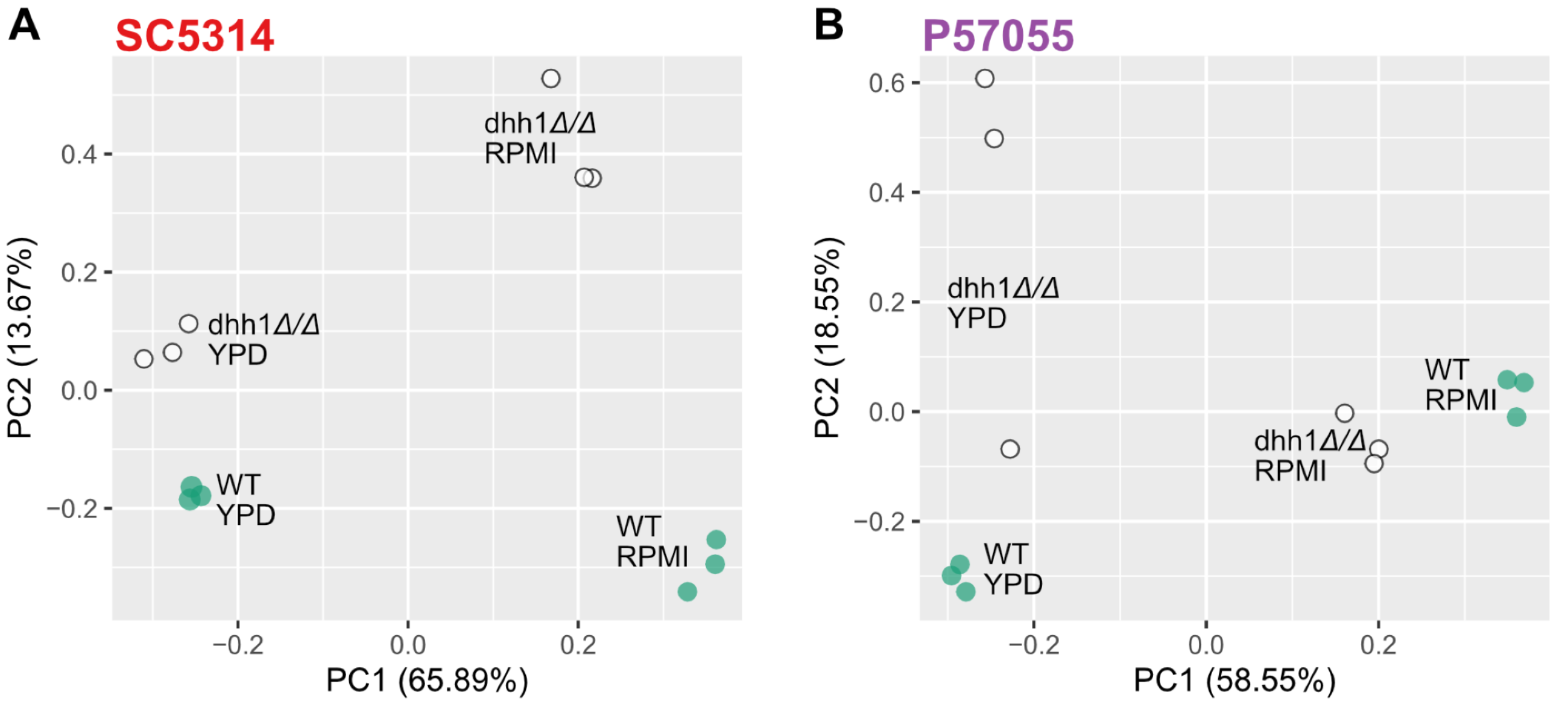

Supplement: S9 Fig — PCA plot displaying the filtered CPM from all 12 experimental groups clustering by condition in A. SC5314 and B. P57055. (TIFF) [file pgen.1011632.s009.tiff]
