# Supplementary material for: Roles of P-body factors in Candida albicans filamentation and stress response
Source: PLoS Genet. 2025 Mar 17;21(3):e1011632. doi: 10.1371/journal.pgen.1011632 (PMC11975087; doi:10.1371/journal.pgen.1011632)
Supplement: Attachment — Submitted filename: Response_to_reviewers.pdf [file pgen.1011632.s024.pdf]

We thank the reviewers for their general appreciation for the manuscript and helpful critiques and suggestions. We revised the introduction, results, and discussion to make the manuscript more concise in addition to expanding the methods for clarity. Most notably, we also characterized the phenotypic effects of *EDC3* and *DHH1* deletion in four additional clinical isolates of *C. albicans* to examine strain heterogeneity. *EDC3* loss had no effect on yeast growth or filamentation across three assays. In contrast, the effects of *DHH1* deletion varied across strains, largely correlating with each strain's propensity for filamentous growth (new Figure 7). We further performed RNA-seq on wildtype and *dhh1Δ/Δ* P57055 to compare the gene regulatory impact of *DHH1* loss in a divergent strain background under yeast and hyphal growth conditions (new Figure 8). This revealed a shared dysregulation of cell wall and metabolic pathways in both strains upon loss of *DHH1*. We included line numbers of our revisions from the clean copy of the revised manuscript in our response. We believe these revisions and additions substantially increase the impact and scope of the manuscript.

Reviewer #1: The work by Tosiano MA et al. investigates the role of P-body component, Dhh1, the DEAD-box helicase involved in mRNA decapping, in filamentous growth of C. albicans. The Authors initially also investigate another P-body component, Edc3, but find no effect of deleting EDC3 on filamentation and focus most of the study on Dhh1. Filamentation is key to virulence of C. albicans. Thus, the subject of this study is significant. The Authors find that cells lacking Dhh1 have aberrant morphology and impaired filamentation. Previous studies implicated Dhh1 and Edc3 in filamentation in C. albicans. This study confirms the involvement of Dhh1 but suggests that Edc3 may not be critical for filamentation. In addition, this study investigates the transcriptome of the cells lacking Dhh1 and finds interesting patterns of gene expression consistent with previous studies in S. cerevisiae which also reveal a hyphae-inducing program in the mutant in non-filamenting conditions. Overall, this is a well-executed and well-described study that thoroughly evaluates transcriptional changes resulting from the deletion of DHH1 gene. It reinforces the role of this gene in mRNA level regulation and the connection to filamentation. This study forms a solid base for future studies that will address roles in gene regulation and filamentation of other specific genes revealed here.

Specific comments/suggestions:

1. Ln 12-14: Are those studies in C. albicans? this should be specified here.

Yes. We revised the manuscript to clarify that the studies pertaining to PB factors and virulence were in *C. albicans* (Ln 13 revised manuscript).

2. Ln 89: perhaps the Authors could explain why specifically these two factors are of interest?

These two factors are of interest for our study because they were both previously implicated in filamentation in *C. albicans*, however their roles in regulating filamentation related genes have only been studied in detail in *S. cerevisiae*. We revised the manuscript to clarify this on revised Lns 80-94,

3. Ln 106-107: this is just one genetic background – perhaps in other genetic backgrounds/strains the Edc3 may have a more substantial role, in contrast to SC5314.

Absolutely! To investigate this, we generated four additional *edc3Δ/Δ* strains in strains representing each of the major *C. albicans* clades. Loss of *EDC3* did not impair colony growth on YPD or filamentation in any of these strains. Our expanded *edc3Δ/Δ* results are presented in Fig. S2 as well as Lns 343-346.

4. Figure 1 D and E - as there is no quantification included, it is unclear if the localizations are unchanged in the absence of the gene or perhaps diminished as compared to the wt

The images for Fig 1. D-E (Fig 1. F-G in revisions) are single plane images, therefore we can not accurately quantify PB features. We agree that the number or size of PBs could be altered with the loss of Dhh1 or Edc3, and note this in the revised methods Lns 539-541. However, we focused on the presence/absence of PBs. We added replicates of the Dhh1-mS, *edc3Δ/Δ* and Edc3-mNG, *dhh1Δ/Δ* strains in Fig S1. B-C to support our conclusion that these factors are not essential for PB formation.

5. Figure 1. DIC/nemarski or an outline of the cell (for confocal) would be helpful.

Thank you for the helpful suggestion, all images in revised Fig 1 have been updated with cell outlines generated with the find edges tool in Fiji on the transmitted light images. This overlays the transmitted and fluorescence light images to show the PBs and cell margins (revised methods Ln 541-544).

6. Figure 1 legend: “10 m at” ; “3 hours of”

We agree and have standardized and revised units of time throughout the paper as the full word.

7. Is it possible that the dhh1 homozygous deletion mutant is an aneuploid or tetraploid?

Formally, it is always possible that *C. albicans* could be aneuploid or tetraploid. However, we believe if this was the cause of the *dhh1Δ/Δ* phenotypes, their phenotypes would not be complemented with WT *DHH1*. Indeed, WT DHH1 fully complements in all five *dhh1Δ/Δ* strains. This strongly suggests the *dhh1Δ/Δ* phenotype we found in five diverse strains is due to loss of *DHH1*, rather than potential chromosomal abnormalities.

8. Ln 225: 75% still remodeled just like in the wt - what is unique/common about those remaining 25% that failed to be remodeled?

Thank you for posing this interesting question. We investigated the genes that were uniquely differentially expressed in WT filamentation, but not in *dhh1Δ/Δ* filamentation in SC5314 using GO analysis. Genes that were only upregulated in WT (and not *dhh1Δ/Δ*) were enriched in nucleic acid decay functions including ribonuclease T2 activity (88-fold enriched) and endonuclease activity (9-fold enriched). Genes that were only downregulated in filamenting WT (and not *dhh1Δ/Δ*) were enriched in amino acid transport (10-fold enriched), one-carbon metabolic process (30-fold enriched), and glycine dehydrogenase (decarboxylating) (60-fold) functions. This supports our model that WT *C. albicans* has a stronger filamentation response than *dhh1Δ/Δ*, increasing nucleic acid turnover and decreasing metabolism. We added this additional information to the revised manuscript at Lns 232-235.

9. Ln 247: the Authors state: "while the overlap of genes upregulated in both conditions was not significant" then later in ln 249 "commonly upregulated genes were involved in... (but this overlap was not significant? – seems contradictory?).

We apologize for this confusion from this section of our original manuscript, which compared the gene regulatory effects of *dhh1Δ/Δ* in YPD to the effects of filamentation in wildtype SC5314. We removed this section from the revised manuscript including the venn diagram in the original Fig. 7 both because it was confusing and to make space for our addition of RNA-seq data from P57055.

10. Fig 8 legend - it should be "yeast-form"

Thank you for finding this. We have corrected the spelling in the Fig 8 legend (revised Fig 9. B)

Reviewer #2: In this manuscript, Tosiano et al. investigated the role of the P-body factor DHH1 in Candida albicans and its impact on filamentous morphology and the transcriptome during filamentation. The authors demonstrate that Dhh1 and Edc3 localized to P bodies, and that P bodies can form independently of Dhh1 or Edc3 in Candida albicans, consistent with the functions of these genes in Saccharomyces cerevisiae. The study also provided evidence that DHH1 is essential for normal growth and WT filamentation behaviors, but that haploinsufficiency does not yield deficiencies in these assays, whereas deletion of EDC3 does not affect growth or heat sensitivity, in contrast to earlier studies on these genes. Complementation of the null phenotypes by the DHH1 gene suggests the present work is correct, and that previous results may be artifacts attributable to the strategy used for deletions in earlier work. Interestingly, deletion of DHH1 resulted in pseudohyphal morphology without the stressors normally required for filamentation. However, in the presence of the triggers for hyphal formation, deletion of DHH1 led to differences in germ tube formation, invasion of media, and biofilm structure. Analysis of the transcriptome of cells lacking DHH1 in yeast-type media showed

differences in gene expression that were similar to WT cells undergoing filamentation, suggesting derepression of expression of some genes in the mutant without stress. In the presence of stress triggers that lead to filamentation, the DHH1 null had additional differences in gene expression. Overall, the work suggests a role for Dhh1 in remodeling the transcriptome to regulate filamentation of C. albicans.

The paper is important and will be of interest to researchers in the RNA/translational control and fungal pathogenesis fields. In general the claims are very well-supported by the data, but there are some minor issues and suggestions that do not require new experiments. In most of the figures, the number of replicates, number of fields analyzed and number of cells for microscopy, significance determinants aren't explicitly indicated. Since the work is in contrast to prior published work, it is critical to show documentation of replicates and significance where used so the strength of claims are clear to the field.

Figure 1-Number of replicates, number of cells imaged need to be indicated, and for 1D and 1E, showing more cells would strengthen the claim that Dhh1 and Edc3 are not independently required for PB condensation. If the authors wanted to make the claim that the factors enhance P-body levels, they would need to quantify granules per cell, but they don't make the claim.

To strengthen our conclusion that Dhh1 and Edc3 are not independently required for PB condensation we added replicates of the Dhh1-mS, *edc3Δ/Δ* and Edc3-mNG, *dhh1Δ/Δ* strains in Fig S1. B-C. Unfortunately, we can not accurately quantify PB granules per cell , as our data for Fig 1. D-E (Fig 1. F-G in revisions) are single plane images (noted in revised manuscript, Lns 539-541). We acknowledge that the number or size of PBs could be altered, with loss of Dhh1 or Edc3. However, for this study we focused on the presence/absence of PBs to investigate the requirement for these factors.

To support the claim that dhh1Δ/Δ cells were twice the size of the DHH1-containing strains, the authors should provide quantifications of cell size from a substantial number of imaged cells, either mean length and width and/or plots of distributions as shown in a latter figure, or mean area -/+ error, so significance of the difference can be evaluated.

Thank you very much for this suggestion. For exponentially grown SC5314 WT, *dhh1Δ/Δ*, and *dhh1Δ/Δ, +DHH1*, we measured the cell and vacuole areas of 100 cells in at least four fields of view. This allowed us to quantitatively compare cell sizes, which are significantly larger in the *dhh1Δ/Δ* strain (new Fig 2. F; Kruskal-Wallis test; $P < 0.0001$). We also evaluated vacuole areas and the percent of each cell filled by the vacuole, which were also significantly increased in the deletion strain (new Fig 2. G-H; Kuskal-Wallis test, $P < 0.0001$) Lns 145-146.

Please indicate the determinant of significance for coloring dots on the volcano plots in Figure 6/7.

Thank you for bringing this to our attention. The colored dots indicated genes with adjusted P values < 0.01 from DEseq2. We added the significance of the colored dots in the volcano plot to the respective figure legends (new Fig 5 and 6) and as well as the volcano plots for P57055 i(new Figs S4 and S5). We also included our P-value cutoff for DEseq2 in our "Transcript alignment, quantification, and quality control" section of the methods (Ln 627-629).

Please indicate what was used for GO term determination in figure 7.

We performed GO enrichment with the *Candida* Genome Database, CGD Gene Ontology Term Finder (http://www.candidagenome.org/cgi-bin/GO/goTermFinder), and have added this to our revised methods section(Ln 634-636).

For figure 6B/DF, please indicate the significance cutoffs used for generating the gene lists for GO and assigning number of gene up/downregulated.

We apologize for the omission. For the GO analyses in revised Figs. 5, 6, S4, and S5, differentially expressed genes with a Log_2 fold-change > +2.5 or < -2.5 (5.6-fold up or down regulated) and Padj < 0.01 from DEseq2. Because fewer genes were differentially expressed in both SC5314 and P57055 backgrounds, we selected transcripts with a Log_2FC > +1.0 or < -1.0 (2-fold up or down regulated) for the GO analysis in Fig. 8. The CGD GO Term Finder reports enriched terms with an adjusted P-value <= 0.1, however the vast majority of terms in our figures had a P-value < 0.01 because we displayed terms with the greatest fold enrichment. We added our methods for GO term determination in the "GO analysis" section of the revised methods (Ln 630-638).

Please indicate in Figures 6F and 7G whether changes in the expression of each transcript in the bar graphs were statistically significant.

Thank you for this suggestion. We added asterisks to indicate significantly differentially expressed transcription factors (Padj < 0.01) in SC5314 in revised Figs 5 and 6. We also used this convention for our new RNA-seq data from P57055 in Figs S4 and S5.

Line 114-nomenclature error

We revised the manuscript Ln 115 to fix the nomenclature issue.

Line 273-the data being discussed don't necessarily indicate RNA decay independent of P bodies, just that there is a P-body-independent function of Dhh1.

We agree with the reviewer and revised the manuscript to "suggesting a PB-independent function in the cytoplasm" Lns 333-334.

Line 280, nomenclature error

Thank you for noting this, we corrected the italics for *DHH1/dhh1Δ* to indicate the heterozygous *DHH1* knockout strain (Ln 340).

Line 282, the authors may wish to remove the contraction for formal writing conventions.

We revised and reworded the entire sentence for appropriate writing conventions on Ln 342-343.

Line 284-extra e

We removed the typo from Ln 344 in the revised version.

Line 286-the data don't report on RNA levels for Edc3, so can't comment on whether Edc3 enhances decapping in this organism, just that it is associated with P bodies. Authors could make the comment more speculative.

Thank you for this excellent point. We added a sentence speculating as to the role of Edc3 in *C. albicans* Ln. 345-348.

Reviewer #3: The manuscript investigates how Candida albicans morphology and stress responses depend on 2 RNA-interacting proteins, Dhh1 and Edc3. These have been investigated extensively in S. cerevisiae, both for their role as P-body markers and in RNA decay. Previous data on the roles in C. albicans was scarce. The major contribution is to show that, in the laboratory strain SC5314, deletion of DHH1 alters filamentation and growth phenotypes and alters the RNA abundance of filamentation-related genes. This is shown several ways and includes complementation of phenotypes dhh1Δ/Δ + DHH1. This observation seems surprising, and as stated contributes to an increasing and important body of "evidence for post-transcriptional regulation of filamentation in C. albicans".

The manuscript is generally reasonably written, and engages with most of the relevant literature including directly comparing their results to published C. albicans datasets. I think that it could be improved by shorter and more focused introduction, RNA-seq results, and discussion, to highlight the main ideas more clearly

We thank the reviewer for their thorough and careful review. As suggested, we have revised the manuscript to be more succinct and focused on the main points of the paper.

The manuscript is lacking in addressing the generalizability of observations to other strains, in validation of the RNA-seq data, multiple problems with the microscopy figure, in some aspects of data presentation, and in inadequate methods descriptions. These limitations need to be acknowledged before acceptance for publication. There are opportunities for new experiments and analyses addressing some of these limitations, that should result in a more impactful manuscript.

We have made extensive additions and revisions to address these issues. We examined the effects of *DHH1* deletion in 4 additional *C. albicans* strains on growth phenotypes (new Fig. 7), and compared the gene regulatory effects of deletion in SC5314 and P57055 (new Fig. 8). We revised Figure 1 to better present our microscopy results and added replicate images in Fig. S1. We also revised our figure legends and substantially expanded the methods section to improve clarity and facilitate reproducibility. By addressing the issues raised by the reviewer, we believe these revisions have indeed resulted in a more impactful manuscript.

Generalizability to other strains. The core observation of DHH1 affecting filamentation of the SC5314 strain appears to be robust, as it's shown by liquid media assays, biofilm assays, and RNA-seq, all in 3-4 replicates. However, there is no evidence as to whether these phenotypes extend to other genetic backgrounds, which is important as extensive recent work from multiple labs argues for the importance of phenotypic variation between fungal strains. It's now known that SC5314 is atypical within C. albicans species complex. One of the authors, Aaron Mitchell, has made the importance of genetic variation a centerpiece of his conference presentations in recent years. The Mitchell lab has published several papers demonstrating that multiple transcriptional regulators in C. albicans have different effects in different genetic backgrounds. These papers include RNA-seq experiments quantifying the importance of genetic background on the results of gene deletion. This body of well-evidenced work argues that the completing the present study to the current standards of the field would involve mutating DHH1 in multiple genetic backgrounds and repeating key phenotyping assays, and ideally include RNA-seq (or otherwise RT-qPCR of a few key targets). If these experiments are not feasible within the authors' resources, they must highlight the major limitation of using only SC5314.

We completely agree. To address this, we deleted *DHH1* in four additional clinical isolate strains representing the major *C. albicans* clades (Hirakawa *et al.,* 2015). Loss of *DHH1* reduced yeast-form colony and planktonic growth in all strains (Fig 2. A-C, new Fig 7. A-B), and this was fully rescued by *DHH1* complementation. Furthermore, after 4 days of growth on YPD plates at 37°C, all *dhh1Δ/Δ* colonies had altered colony morphology regardless of genetic background (Fig 2. D, new Fig 7. C). We also carried out new RNA-seq experiments to compare the effects of *dhh1Δ/Δ* in strains SC5314 and P57055 under yeast and early hyphal conditions (Fig. S4-S5; new Fig. 8). This showed that most, but not all, of the regulatory changes are consistent in the two strains, which both validates our results and helps refine the common pathways affected by *DHH1* loss. We also deleted *EDC3* in these clinical isolates, and determined that its loss has no significant growth impairment, as we originally observed in SC5314 (Fig. S2).

The discussion acknowledges a related limitation, regarding condition-dependent filamentation: "These apparently diametrical results are not uncommon in other C. albicans genetic backgrounds where the same strain may have varied filamentation phenotypes under different conditions." I agree that extending the work to other filamentation conditions, in addition to other strains, would increase the impact of the work.

As suggested, we expanded our spider plate filamentation assay to four additional WT and mutant strains. We believe the common defects we observed firmly establishes the general importance of *DHH1* in *C. albicans* filamentation. As such, we did not perform additional filamentation assays in these 4 new backgrounds. However, we added language to the new paragraph on strain heterogeneity in the revised discussion to bring

this to readers' attention Ln 353-354. While we agree this is an interesting area for future research, we believe additional filamentation conditions would be beyond the scope of the current study.

Validation of the RNA-seq data, at least, testing of key biological hypotheses arising from the data. The current manuscript ends with the RNA-seq analysis, whose main results are that loss of DHH1: 1. disrupts pathways involved in metabolism and morphology, including leading to upregulation of filamentation pathways in otherwise non-filamenting conditions, and 2. reduces filamentation dependent transcriptome remodeling. This first result is striking. The manuscript presents these results at length and discusses around 2 hypotheses: "the loss of DHH1 not only impacts the direct targets of mRNA decay, but downstream regulation of the yeast-to-hyphae gene regulatory network". Is there any way that direct vs indirect effects could be tested? Any hyphal regulator that could be over- or under-expressed to suppress the effect of dhh1Δ? Any direct targets of DHH1 that could be manipulated to test if this interaction is causative? Any S. cerevisiae DHH1 datasets (CLIP-seq from Mitchell et al., 2013; RIP-seq from Miller et al., 2018) that could be analysed to directly compare targets? As it is, it feels like the manuscript stops and speculates just after the most interesting observation, and any steps the authors could take to move the story forward here would increase the impact of the observation.

We agree that further studies of the *DHH1* regulon are an exciting area for additional research. Because *DHH1* deletion perturbs the expression of thousands of genes, determining which effects are direct, and which are indirect, will be challenging. Although we appreciate the reviewer's suggestion to compare our results to *DHH1* CLIP- and RIP-seq studies from *S. cerevisiae*, we believe such a comparison is unlikely to be helpful. The two species diverged ~ 300M years ago (Hedges *et al*., 2015), and their gene regulatory networks have undergone extensive rewiring (Dalal *et al*., 2016). In addition, our experimental conditions differ substantially from those of Mitchell *et al*. and Miller *et al*. As such, it would be difficult to interpret such a comparison. An alternative way to identify core regulatory targets is to examine the effects of DHH1 deletion that are shared across clinical isolate strains (Huang *et al*. 2019). We believe our added comparison of RNA-seq data is a good starting place, and will help to provide a rationale for future study of the DHH1 regulon.

Microscopy data in figure 1 has multiple problems, that could be easily addressed. It shows only stress conditions, acute heat shock (46°C 10 min) or nutrient starvation ("3 hours of acute glucose starvation"). Non-stress conditions need to be included so that readers can judge if there is a difference. The glucose starvation condition was not clearly described in methods.

We agree. To help readers judge this, we added a non-stress condition as Fig 1. A (30°C and complete media). We also added information on the glucose starvation conditions in Fig 1. D in the figure legend and the revised methods (Ln 495-496).

Panels 1D and 1E the conditions aren't clear. There's no quantitative analysis of P-bodies/foci, and it would be very nice to know how many there are per cell or if either Edc3 or Dhh1 affects the number of foci of the other.

We apologize for the lack of clarity. We added the acute heat shock conditions of Dhh1-mS, *edc3Δ/Δ* and Edc3-mNG, *dhh1Δ/Δ* in Fig 1. F-G in the figure legend (CM, 10 minutes at 46°C) and included additional information in the Methods section under "Phenotype assays" (Ln 490-498).

While we agree it would be interesting to examine the number of PBs in wildtype and mutant cells, for this study we focused on whether the factors were essential for PB formation, as had been previously reported (Jung and Kim 2011). As such, we assayed the presence/absence of PBs, To further support our conclusion that Dhh1 and Edc3 are not independently required for PB condensation we added replicates of the Dhh1-mS, *edc3Δ/Δ* and Edc3-mNG, *dhh1Δ/Δ* strains in Fig S1. B-C. In addition, our data collected for Fig 1. D-E (revised Fig 1. F-G) are single plane images, therefore we can not accurately quantify the number of foci per cell. However, we added language on Lns 539-541 acknowledging that the number or size of PBs could be altered, with loss of Dhh1 or Edc3.

Also, this figure is disconnected from the rest of the manuscript: what about during the YPD and filamentation conditions used in later figures?

Unfortunately, the conditions used for yeast (YPD at 30°C) and filamentous growth (RPMI + 10% FBS at 37°C for 4 hours) later in the manuscript are not compatible with fluorescence imaging. YPD and FBS have a large amount of autofluorescent background which preclude imaging with our fluorophores. We added new images of PB formation in filamenting SC5314 over a 4 hour time course in RPMI minus phenol red at 37°C in Fig 1. E, which is similar to conditions used for filamentous planktonic growth and RNA-seq. We note that we had to omit FBS from this condition (Lns 124-125, 448-449, 496-497) which may be a somewhat milder filamentation condition.

Does Dhh1 forming P-bodies matter for its effect on morphology and gene expression?

This is an interesting question. Work in *S. cerevisiae* has demonstrated that Dhh1's ATPase activity is needed for translocation between the cytoplasm and PBs (Carroll *et al*., 2011, Mugler *et al*., 2016). In *S. cerevisiae*, ATPase defective Dhh1 accumulates in PBs due to slowed disassembly, and has slower turnover of transcripts and causes heat sensitivity (Carroll *et al*., 2011, Mugler *et al*., 2016). Therefore, we expect that accumulation of Dhh1 in PBs could impact heat tolerance and filamentation in *C. albicans*, possibly due to reduction of cytoplasmic Dhh1. We have added language to the revised Discussion (Lns 333-338) to raise this interesting question for readers.

Data presentation is most of an issue for the RNA-seq where the description is patchy and not always in a helpful place for the reader. The discussion of the DGE analysis and GO enrichment is thorough, but the information needed for an RNA-seq expert to assess or reproduce the experiment is inadequate. Could the number of replicates and basic QC be mentioned in the results in addition to the bigger description in methods?

Certainly. We added these details to the revised results section and the expanded "RNA-extraction and sequencing" and "Transcript alignment, quantification, and quality control" sections in the revised materials and methods (Lns 572-575, 609-620, & 621).

How were RNA-seq libraries prepared - poly(A) enrichment or rRNA depletion, which kit? What was the depth of sequencing?

We apologize for omitting these important details from the original submission. mRNA-seq libraries were prepared using a NEBNext Poly(A) mRNA Magnetic Isolation Module (NEB #E7490) and the NEBNext Ultra II Directional RNA Library Prep Kit for Illumina (NEB #E7760S/L). Our read depth ranged from 18.6 million to 37.5 million aligned reads (average depth of 26.6 million). We revised the methods section to include this information.

Can the analysis pipeline be described more clearly, the methods aren't totally clear if edgeR or DESeq2 were used or both? Which alignment settings for HISAT2, which genome release, was the haploid or diploid genome used? Can all counts per gene in tabular format be added to NCBI GEO as well as the raw counts on SRA? The descriptions of growth conditions and sample preparation from the author link at SRA are also incomplete.

Yes. We expanded the "Transcript alignment, quantification, and quality control" section of the materials and methods to comprehensively describe our RNA-seq analysis pipeline. DESeq2 was used for the differential expression analysis (Ln 627-629). The parameters for HISAT2 alignment included splice sites, strandedness, a maximum intron length of 1400 and aligned the hits to the diploid R22 genome (Ln 609-610). We also updated the metadata for the SRA accessions and created a GEO submission with the raw counts for reads mapped to genes. We added those data to new supplemental tables S10-11 as well.

The data presentation for figures 3-5 (morphology) was overall ok. Figure 3A should add replicate or error bar information. In figure 3 It would be nice to have quantification of vacuole size given that's a conclusion. The quantifications in figure 4-5 were effective.

Thank you for this suggestion. For growth in liquid YPD, we repeated the experiment with three replicates for each strain (WT, *dhh1Δ/Δ,* and *dhh1Δ/Δ, +DHH1*) and included error bars showing the 95% confidence interval (revised Fig 2. C and Fig 7. B). We also added quantification of the cell and vacuole sizes and calculated the area of the cell taken up by the vacuole (Percent Vacuole Area; PVA). The median PVA was roughly three times larger in *dhh1Δ/Δ* than in *DHH1* containing strains (Fig 2. F-H.; Kruskal-Wallis test; $P < 0.0001$) Lns 145-146.

The methods overall need extensive additions and clarification, e.g. are these whole-ORF deletions, what linkers were used for fluorescent tagging, what were the PAM sites, numbers of replicates/labware/volumes/method of OD recording for growth curves, details or reference for acid phenol RNA extraction, parts numbers for kits and enzymes, how were images processed beyond "with Fiji", and many others.

We apologize for these omissions. We extensively revised our “Materials and methods” section to address these issues. Our fluorescent tagging constructs include a GGSG linker (Frazier *et al*., 2019). Our methods for whole ORF deletions of *EDC3* and *DHH1*, and *DHH1* complementation are clarified in Lns 453-464 and the new Fig. S8. Our PAM sites are listed in the new Table S9. We also added details for growth curves in YPD (Lns 502-505) and RNA extraction methods including kits and enzymes (Lns 571-607). Details on image processing in Fiji for our phenotype data are clarified on Lns 533-570 as part of the “Imaging and processing” section. In general, we revised the materials and methods for clarity and to facilitate reproducibility.

Given the need for major clarifications and revisions, I did not go through line-by-line to suggest minor revisions.

To end: the observation that Dhh1 affects filamentation is interesting, and the wider point of the importance of post-transcriptional regulation in fungal morphology and virulence is an important direction for the field. There's good work here and some solid observations upon which to build a stronger revised manuscript.

Once again, we thank the reviewer for their thorough consideration of our manuscript. We believe that, by addressing the issues they raised, the revised manuscript is much improved and will be well received by PLoS Genetics readers.

Have all data underlying the figures and results presented in the manuscript been provided?
Large-scale datasets should be made available via a public repository as described in the *PLOS Genetics* data availability policy, and numerical data that underlies graphs or summary statistics should be provided in spreadsheet form as supporting information.

Reviewer #1: Yes

Reviewer #2: Yes

Reviewer #3: **No:** Counts per gene for RNA-seq (but, I had trouble accessing the supplemental data files due to traveling while writing my review, so did not check underlying data for all other figures)

In addition to our DESeq2 results in Tables S1-S4 and S5-S8, we added our raw RNA-seq counts per gene in SC5314 and P57055 as new Tables S10-S11. The raw RNA-seq counts per gene have also been added in a new submission to the NCBI GEO.
