# Supplementary material for: Roles of P-body factors in Candida albicans filamentation and stress response
Source: PLoS Genet. 2025 Mar 17;21(3):e1011632. doi: 10.1371/journal.pgen.1011632 (PMC11975087; doi:10.1371/journal.pgen.1011632)
Supplement: Attachment — Submitted filename: Response_to_reviewers_Rev2.pdf [file pgen.1011632.s025.pdf]

We thank the reviewers for their time and expertise. Your comments have greatly strengthened the manuscript allowing us to make a greater contribution to the field.

Reviewer #1: The Authors have addressed all of my comments adequately.

We thank Reviewer #1 for their time and attention to detail. The comments suggesting generating *edc3Δ/Δ* in multiple WT background strains and revisions for PB imaging in Figure 1 were particularly helpful.

Reviewer #2: The new experiments showing effects of DHH1 on filamentous growth/colony morphology, temperature tolerance, and RNAseq (for one isolate) of clinical isolates strengthen the claim that DHH1 is critical for WT responses to clinically relevant stressors and increases the impact of the work. It is interesting that the clinical isolates are more sensitive to DHH1 deletion for growth rates and temperature sensitivity, suggesting a critical role for DHH1 in the hyperfilamentous growth phenotype of these isolates.

The authors have addressed all of my comments in the revision and I would recommend publication of the revised manuscript.

We thank Reviewer #2 for their helpful suggestions in regard to clarifying language and significance values. Additionally, quantifying the size of *dhh1Δ/Δ* cells in Figure 2 greatly strengthened our evidence for loss of DHH1 generating larger stressed cells.

Reviewer #3: The authors have thoroughly responded to reviewer comments, adding new data, quantifying existing data, and strengthening the manuscript with effective rewriting. It is really a pleasure to see reviewers giving consistent and constructive feedback, and authors responding to this so strongly.

Addition of the extra strains substantially strengthens the manuscript, showing that phenotypes are consistent across strains with underlying variation. Figure 7 is very nice indeed and presents the data effectively, as does Figure S2 and Figure 9A. The argument in the discussion is very nice, that dhh1-deletion changes suggest an “identity crisis”. Still, could the manuscript more clearly discuss why they chose these other 4 strains out of all strains? Especially why they did the additional RNA-seq in P57055 only?

We thank Reviewer #3 for their extensive comments. Your input has significantly strengthened this manuscript’s contributions to the field. We clarify in Ln 256 and Lns 282-284 that we chose to use the four additional strains that we previously used for gene deletion studies, as these represent a broad genotypic and phenotypic diversity, and that we performed RNA-seq experiment in P57055 due to its phylogenetic distance from SC5314 (second most distant strain of the 4) as well as its similar WT filamentation capacity and *dhh1Δ/Δ* phenotype aberrations.

Some minor comments.
Line 138, "After 3 hours of acute glucose starvation in PBS" - this could be further clarified, because 3hrs in PBS is starvation for everything else as well as glucose.

We updated this from “acute glucose starvation” to “acute starvation” to clarify.

Line 460, formatting problems in "EDC3mNeonGreen and DHH1mScarlet tags"
Line 536, "SC5313 background"

We fixed these typos.

Supplemental information spreadsheet Tables S1-S13 - it would be great to add a sheet zero with the table of contents for this in the same file, to aid navigation.

We added a Table Index to the supplemental file.

Additional Editor Comments :

Journal Requirements:

1) Thank you for stating that "Raw RNA-seq data have been deposited at the NCBI SRA under bioproject number PRJNA1133750. Processed expression data are available at NCBI GEO under accession GSE284543". Please note that, though access restrictions are acceptable now, your entire minimal dataset will need to be made freely accessible if your manuscript is accepted for publication. This policy applies to all data except where public deposition would breach compliance with the protocol approved by your research ethics board. If you are unable to adhere to our open data policy, please kindly revise your statement to explain your reasoning and we will seek the editor's input on an exemption.

We updated the release dates on these data.

2) Please upload the figures in a correct numerical order in the online submission form.

We have done this.
